# Do Adolescent Developmental Issues Disappear Overnight? Reflections about Holistic Development in University Students

**DOI:** 10.1100/tsw.2011.5

**Published:** 2011-02-14

**Authors:** Daniel T. L. Shek, Keri K. Wong

**Affiliations:** ^1^ Department of Applied Social Sciences, The Hong Kong Polytechnic University, Hong Kong; ^2^ Public Policy Research Institute, The Hong Kong Polytechnic University, Hong Kong; ^3^ Kiang Wu Nursing College of Macau, Macau, China; ^4^ Division of Adolescent Medicine, Department of Pediatrics, University of Kentucky College of Medicine, Lexington, KY, USA; ^5^ Centre for Family Research, University of Cambridge, UK

**Keywords:** mental health, developmental issues, positive youth development, prevention programs, university students

## Abstract

Adolescent developmental issues, such as mental health problems, substance abuse, and egocentric behavior, of university students are examined. This conceptual review generally shows that although there are related issues among university students deserving greater attention, there is a general lack of systematic prevention or positive youth development programs adopting the principle of universal prevention. In contrast to the abundance of universal adolescent prevention and positive youth development programs specifically designed for high school students, similar programs are grossly lacking in the university educational context. This paper highlights the factors contributing to such negligence in university education and the possible strategies that can be adopted to help university students develop in a holistic manner.

## INTRODUCTION

Adolescence is a period of transition when intense changes in different domains take place. With reference to the growing adolescent developmental issues, such as substance abuse, gambling, and delinquency in high schools, there are adolescent prevention and positive youth development programs specifically designed for high school students. With regard to adolescent substance abuse, a review of the programs at the Centre for Substance Abuse Prevention, Substance Abuse and Mental Health Service Administration, Department of Health and Human Services, the U.S. Government showed that there are hundreds of programs developed for high school students and some of them have been shown to be effective in reducing adolescent substance abuse[1].

Logically speaking, when high school students enter college, it is expected that developmental issues observed in high school (e.g., substance abuse, depression, poor psychosocial competencies, etc.) will not disappear overnight. As such, it is important to ask how university education could, would, and should help university students, normally in their late teens and early twenties, to thrive. In particular, it is important to ask how risk behavior and positive youth development are addressed in university learning, especially in the formal curriculum. Against this background, this conceptual paper examines how developmental issues in university students should be addressed and how holistic development in university students could be promoted. There are several parts in this paper. First, adolescent developmental issues, particularly mental health issues, among university students are examined. Second, issues related to the "dark side" and needs of university students (such as geocentricism, lack of integrity) are reviewed. Third, arguments for and against possible options for nurturing university students are explored. Finally, the possible role of credit-bearing courses on positive youth development in addressing adolescent developmental needs is discussed.

## MENTAL HEALTH ISSUES

According to Chen et al.[2], the university years constitute a stressful time of change for students. Students begin to fine-tune and master skills to adapt to and withstand new challenges. They also gain self-understanding of their identity, and their sense of purpose and goals, through exposure to a multitude of experiences and opportunities. To graduate from college is to persevere through the frequent bombardments of psychosocial stressors (e.g., examination and financial stresses). The increasing competition due to rising admissions standards, tuition costs, and cutbacks on student loans, in addition to the academic and psychosocial stressors at college, marks the plight of college campuses today[3]. According to the latest Higher Education Research Institute report[4], the primary concern for freshmen is the ability to fund their college education, in which two-thirds of students reported financial burdens being "some" or a "major" concern (66.7%). By not addressing these problems and concerns, we are doing a great disservice to the students, the future leaders of our society. Hence, the need to promote well-being among college students and to abate reciprocating harmful effects for our society has become an urgent one.

In addition to the psychosocial stressors, studies show that mental health is a growing concern among university students. Blanco and colleagues[5] assessed the 12-month prevalence of psychiatric disorders, sociodemographic correlates, and rates of treatment among college (n = 2,188) and noncollege attendees (n = 2,904) in the U.S. He found alcohol use and personality disorders to be the two most prevalent disorders across campuses, at 20.4 and 17.7%, respectively. College students relative to noncollege attendees were at greater risk for alcohol use, but were less likely to use drugs and nicotine, be diagnosed with bipolar disorder, and to receive treatment. Approximately half of all respondents met the DSM-IV criteria for at least one psychiatric disorder in the past year, regardless of education status. As remarked by Mowbray et al.[3], "averaging across a number of studies, it appears that approximately 12–18% of students on college campuses have a diagnosable mental disorder" (p. 227).

Depression is the leading instigator to a variety of negative outcomes contributing to the bulk of suicides on college campuses. A web-based survey of 2,843 Midwestern undergraduates found a prevalence rate of 15.6% for depressive or anxiety disorder[6]. In another study, clinical anxiety was found in 46% of male and 64% of female respondents, while clinical depression was found in 12 and 15% of the same groups, respectively. Of those entering college, approximately half (49%) will become severely depressed during the course of college[7].

Suicide is the second leading cause of death among college students in the U.S. after homicides and accidents[8,9]. According to the Big Ten Colleges Study, the death rate of suicide among college students is at an astonishingly high rate of 7.5/100,000[10], with lasting immeasurable damages for individuals acquainted with the victim (e.g., family, friends). According to the 2009 American College Health Association's National College Health Assessment[11] of 2000–2008 spring surveys of 106 private and public institutes, 93.7% of the 80,121 students reported feeling "overwhelmed" in the past year, with 43% feeling so depressed that it affected functioning. Nine percent of respondents "seriously considered attempting suicide" and 1.3% actually "attempted suicide" in the past year. These findings are consistent with the Furr et al.[12] survey of 1,455 college students from four universities, where 53% of students reported some form of depression while at college, 9% reported they had thought of committing suicide, and 1% reported a suicide attempt.

Another major concern for campuses today is the issue of drug abuse and alcoholism[13]. As of 2008, narcotic use was the third most prevalent substance use on campuses, following closely behind alcohol use[14]. The 2003 National Center on Addiction and Substance Abuse (CASA) report entitled "Formative Years: Pathways to Substance Abuse among Girls and Young Women Ages 8–22", conducted by Columbia University[15], examined substance abuse among girls and young women, and found that the transition into college is when the greatest increase occurs in smoking, drinking, and marijuana use. Researchers showed that roughly 40% of university students had heavy episodic drinking and 20% were diagnosed as having alcohol abuse or dependence in the past 12 months[14,16]. Despite the worrying trends of college mental health, only around one-tenth of the students who experience psychosocial problems actually seek help.

## THE "DARK SIDE" OF COLLEGE STUDENTS

College students today, also known as the Me Generation or the Millennials (born 1981 to present), are found to be distinctly different from previous generations. Although they are team-oriented, confident, independent, ambitious, and highly motivated[17], they are not empathetic. A recent study presented at the Association for Psychological Science Annual Convention 2010 observed a sharp decline in empathy among college students after the year 2000[18].

The finding that there is a gradual drop in empathy among college students is consistent with previous reports of rising narcissistic personalities. There are research findings showing that narcissism levels in U.S. university students have gradually increased over the past 25 years[19], with increases seen in management[20] and business students[21]. Twenge et al.[19] compared mean narcissism scores during 1979–1985 and found almost two-thirds of contemporary university student scores to be above the mean. There is an overall increase in narcissism across the cohort today[22,23]. As a general observation, graduates today are more concerned about "how valuable they are" rather than "how they may be valuable." Compared to previous generations, students are more self-interested rather than team-orientated and in general more driven, yet more stressed, to meet the expectations of authorities (i.e., parents, employers)[24] and more inclined to seek rapid promotion and acquire new skills at their jobs[25].

A recent New York Times article titled "*Students are Different Now"* discusses that "students now are less mature and often not ready for the responsibility of being in college" and that "many students today lack the resilience and are unable to summon strategies to cope." Acknowledging the economic stress and political realities that this generation faces, college experience itself is not thought of as more stressful than 10 years ago, but that "many students are often not prepared to be young 'adults' with all the responsibilities of life"[26].

Over the years, students' pattern of civic engagement and social responsibility may have changed in nature. Sax[27] observed a decline in political interest over the last decade, contrary to volunteerism, which has steadily increased since 1990. Contrastingly, Kiesa and colleagues[28] surveyed 12 4-year colleges and revealed that Millennials were actually more engaged in the community and politics than their parent's generation, but in a broader spectrum of community experiences than in previous years. Also supporting this claim is the recent Higher Education Research Institution (HERI) report of national norms for the freshman class of Fall 2009[4]. Again, volunteerism has reached a record high; with intended participation in community service being 30.8%, and 6.3% stating no intention of volunteering while at college, which was a substantial increase in interest over the last 2 decades when the interest was around 17% (16.9%). There were also several substantial declines in students' attitudes in Fall 2008, including a decline in interest about political affairs (3.5%), the importance of promoting racial understanding (4.2%), developing a meaningful philosophy of life (3.4%), and influencing social values (2.6%). In terms of students' goals, the top two were to be financially well-off (78.1%), the highest figure since 1966 when the question was first asked, and raising a family (74.7%) following closely behind. As evident, these findings may reflect a shift in values among college students, to a more self-focused and financially oriented philosophy, which is in line with a decrease in social responsibility and civic participation.

Loeb[29] observed and surveyed college students across 30 states in the U.S. throughout the late 1970s and early 1990s, and noted that they were "pathologically selfish, greedy, apathetic, and unconcerned with higher ideals" (p. 2). He believed that students' values have changed. When comparing student activists with the passive apathetic peers, several common beliefs surfaced, such as "the world was inherently unfair, and that they had little possibility of changing it. Buried by outside jobs and by massive debt loads, threatened by a dubious economic future, "these students feared they were on their own in terms of personal survival" (p. 3). To Loeb, students' fears were not solely of the economy, but a product of the cultural climate that reinforces personal gains over group causes. Loeb further questions the extent to which students should remain passive, fearful, apathetic citizens, or active, engaging leaders of America.

## HOW SHOULD UNIVERSITIES RESPOND?

Although comparative data on mental health problems and "dark-side" attributes among university students are almost nonexistent, available research findings are strong enough to demand that university administrators and educators ask an important question – How can we help university students to develop in a balanced and holistic manner? Currently, higher educational institutions use three methods to attenuate problems of mental health and to promote holistic development on campus, but they are not without flaws. First, designated university departments, such as the Student Affairs Office (SAO), operate with the purpose of providing students with the resources and services to develop important nonacademic skills (i.e., leadership skills, executive planning, interpersonal skills, social responsibility, etc.). SAOs strive to "facilitate the development and growth of students of the University by providing relevant and quality services and support"[30] or act as "a significant contributor to students' all-round development"[31]. Each student is entitled to a range of services from career planning and development, to leadership training and extracurricular activities, to the promotion of psychological well-being and methods of coping with psychosocial stress at college. In some cases, this means health care and counseling services, and even same-day appointments for severe cases of depression[32]. Services provided by SAOs equip students with useful, transferable skills for them to live a meaningful and fulfilling life, which complements their formal educational training. However, it is noteworthy that only those who proactively seek SAOs, which is a minority, benefit from the outcomes.

The second possible strategy is curriculum infusion (CI), which refers to inserting health-related information, such as substance abuse prevention content, into existing college courses while simultaneously meeting the course objectives. There are studies suggesting that CI was effective in reducing students' desire for use of drugs and binge drinking, as well as informing them about campus resources[33,34,35]. While CI is an innovative approach that combines adolescent developmental issues and formal curriculum (so that no student would miss it), its effects depend very much on the enthusiasm and personal qualities of the instructors. In addition, it can be argued that this strategy mainly focuses on adolescent problems instead of promoting positive youth development.

The third possible option is to design credit-bearing courses on adolescent developmental issues and positive youth development. There are several advantages to this. First, it is consistent with the spirit of the public health approach that universal prevention is important. Second, as dosage of the intervention is positively related to outcomes in adolescent prevention programs, the use of credit-bearing courses, which usually involve more than 10 h for a 1-credit course, can ensure that sufficient time and effort are spent on the related adolescent concerns. Finally, the involvement of credits can enhance the motivation of the students.

Of course, there are several arguments against the use of credit-bearing prevention or positive youth development programs, particularly when such courses are compulsory in nature. First, one may argue that the curriculum is so dense already that there is no room for such courses. Second, one may argue that students should be given the freedom to choose their own courses in the university settings and it is not appropriate to have compulsory requirements. Third, one may argue that it is technically not feasible because there are not enough qualified trainers for such courses.

There are several counterarguments to refute the above arguments. Regarding the query of a dense curriculum, it begs the question of what is our educational vision? It is important that we should always create space for meaningful subjects. Concerning the issue of freedom, it is noteworthy that there are, in fact, compulsory subjects in the area of languages and General Education that students have to take. Therefore, it is the question of educational vision again. Finally, if validated adolescent prevention and positive youth development programs are developed and well-designed training programs are available, the question of inadequate trainers can be solved. As adolescent developmental issues do not disappear overnight (i.e., they continue from high schools to universities), university administrators and educators must rethink the question of how university education, particularly the formal curriculum, can help to promote adolescent development.

Unfortunately, a review of the literature shows that credit-bearing adolescent positive youth development courses are very few. To our knowledge, few (if any) college courses prepare students with the nonacademic “life skills” outside of their formal education; namely, a credit-bearing course that emphasizes social responsibility, mental health awareness, and leadership skills for college students of all concentrations. Courses that foster holistic student development are distinct in nature in that some are offered as mandatory requirements for majors[36], free electives for those with an interest[37], or extra credits toward the final course grade[38].

Credit-bearing courses that promote students’ holistic development are more common for students in occupations that require social interaction and experiential learning. Shapiro[36] placed 20 first-year undergraduate Gerontology Honors students at community agencies in which they served elders nearing death for a minimum of 4 h/week. In addition to coursework, students engaged in weekly discussion, reflected on their service experiences through an argumentative paper, peer reviewed, and submitted a creative virtual scrapbook as a class for the final assignment. Overall, students responded positively after engaging in the service with four out of 20 students continuing with their placements the following semester. Similarly, Deeley[39] conducted a qualitative study investigating the positive and negative effects of service learning on students of public policy majors. Several themes emerged from service participation, such as positive improvements in confidence, critical reflection, experiential learning, personal transformation, challenges during placement, and feelings of discomfort upon realizing their own changes.

Obviously, the development of credit-bearing courses to promote holistic development in university students is not without controversy. Pickles[40] discussed the existing plight of higher education in the U.K. The controversy concerned whether such courses should be embedded into the curriculum, offered independently, or as a nonacademic resource where the same goals are reached. The strengths of having a course embedded into the curriculum are that students are likely to be competitive candidates to employers (more confident, mature, and competent) and weaknesses are the foregone time available for the content of the subject matter, and it will be tough for students who wish to truly immerse themselves into their subject of interest. Pickles reiterated several times the importance of nurturing “better learners with a greater awareness of their individual needs, including the need to develop habits that will lead to lifelong learning.” (p. 2). When considering the possibility of having credit-bearing courses, it is worth considering the comment by Eisen et al.[41], “we and others wondered whether the original integrated goal of a liberal education — a vision balancing knowledge and discovery with personal well-being and civic engagement — has been forgotten by overemphasizing knowledge and splintering student life into so many noncommunicating fiefdoms. If so — and if health is more than merely the absence of disease and includes mental, physical, and spiritual well-being — then this has been done at the expense of student health and healthy living" (p. 455).

## EXPERIENCE IN HONG KONG

In an age of accountability, educators are more concerned about student outcomes. Unfortunately, while outcomes in tertiary education are commonly related to academic and occupational domains, university administrators only pay lip service to holistic development in young people. As argued provocatively by Astin and Sax[42], "although we argued that institutions needed to focus more on student outcomes, we avoided specifying what any of these outcomes should be, arguing instead that this task should be left largely to the individual institution. In retrospect, I think this was a mistake. If we had been more forthcoming about our own values with respect to some of the most important student outcomes, we certainly would have generated more controversy, but I think the controversy would have been healthy. More specifically, I wish we had spoken more directly about the importance of so called affective outcomes such as self-understanding, tolerance, honesty, citizenship, and social responsibility" (p. 587).

Shek et al.[43] identified many developmental issues and concerns in university students in Hong Kong. First, research and statistics show that there are several developmental issues in young people in Hong Kong, such as growing adolescent substance abuse and worsening of mental health of young people. Second, research findings suggest that poor mental health among university students is an issue deserving attention. Third, findings based on employer surveys commonly showed that employers in Hong Kong were not satisfied with the personal qualities of graduates in the areas of interpersonal cooperation and maturity. Fourth, although schools in Hong Kong commonly claim to emphasize "whole person" development, this emphasis is not reflected in the formal curriculum. Finally, different advisory bodies of the Government of the Hong Kong Special Administrative Region are actively emphasizing the importance of nurturing qualities in young people.

Against such a background and adopting the argument that adolescent developmental issues do not disappear overnight, Shek et al.[43] argued that the development of credit-bearing courses on positive youth development would be helpful in order to nurture university students and proposed several principles in the development of such courses. These included holistic student development, responding to community concern about young people, preparing students for adulthood and general education with life-long benefits, uniqueness, universal coverage, theory-driven general education programs, and research-driven general education programs. As far as the model of positive development is concerned, Shek et al.[43] argued that universities should help to develop 14 positive youth developmental assets identified by Catalano et al.[44] based on effective positive youth development programs. The utilization of these constructs in course design is consistent with the spirit of evidence-based practice. The constructs are as follows:

1. Promotion of Bonding: Developing program participants' relationship with healthy adults and positive peers in the extrafamilial contexts (school, community, and cultural contexts), and caregivers and significant others in the intrafamilial context.
2. Cultivation of Resilience: Promoting the capacity for adapting to change and stressful events in healthy and adaptive ways.
3. Promotion of Social Competence: Training program participants' interpersonal skills (including communication, assertiveness, refusal and resistance, conflict resolution, and interpersonal negotiation) and providing opportunities to practice such skills.
4. Promotion of Emotional Competence: Training program participants' skills to recognize feelings in oneself and others (including empathy), skills to express feelings, skills to manage emotional reactions or impulses (delay of gratification and frustration, tolerance), and emotional self-management strategies.

5. Promotion of Cognitive Competence: Developing program participants' cognitive abilities, processes, or outcomes, including academic performance, logical thinking, critical thinking, problem solving, decision making, planning and goal setting, and self-talk.
6. Promotion of Behavioral Competence: Cultivating verbal communication (making requests and positive response to criticisms), nonverbal communication, and action-taking skills, and providing reinforcement for the effective behavior choices and action pattern.
7. Promotion of Moral Competence: Developing a sense of right and wrong, and respect for rules and standards as well as social justice.
8. Cultivation of Self-Determination: Promoting program participants' sense of autonomy, independent thinking, or self-advocacy.
9. Promotion of Spirituality: Helping program participants to develop purpose and meaning in life, hope, or beliefs in a higher power.
10. Development of Self-Efficacy: Promoting program participants' coping and mastery skills, and changing their negative self-efficacy expectancies or self-defeating cognitions.
11. Development of Clear and Positive Identity: Promoting healthy identity formation and achievement, including positive identification with one's social or ethnic identity.
12. Promotion of Beliefs in the Future: Helping program participants to develop future potential goals, choices, or options.
13. Providing Opportunities for Prosocial Involvement: Designing activities and events for program participants to make positive contribution to groups.
14. Fostering Prosocial Norms: Encouraging program participants to develop clear and explicit standards for prosocial engagement.

The two courses described in Shek et al.[43] have been approved to be General Education courses by The Hong Kong Polytechnic University. The first course on leadership will be piloted in 2010–2012, and systematic teaching materials on self-understanding and interpersonal communication will be developed. However, we do not argue that the course on self-understanding and interpersonal development by itself is adequate enough to promote positive development in university students. Actually, there are research findings showing that other courses, such as service learning, can help university students to develop mature behavior and civic responsibilities. However, in carrying out service-learning tasks, students should have some basic qualities such as self-understanding and interpersonal communication skills in the first place. Therefore, one should realize the intimate link between positive youth development and other student development programs, such as service learning.

In addition, as credit-bearing courses are proposed for promoting positive youth development, it is important that the courses be put in the learning context and "effort" alone is not sufficient to achieve the learning outcomes. Assessment methods reflecting both intellectual and personal development of the students are, in fact, equally important. Finally, as in other programs, one should be conscious of the possible unintended consequences of the related programs. While the positive youth development constructs reviewed were derived from the effective programs in the literature, which did not show unintended negative consequences, such programs are mainly designed for high school students. With the use of different evaluation strategies, we will then be able to understand the effectiveness of the related courses in promoting the holistic development of university students.

In response to the comment of Chickering[45] that colleges and universities "have generally ignored outcomes related to moral and ethical development as well as other dimensions of personal development" (p. 1) and "have failed to graduate citizens who can function at the levels of cognitive and moral, intellectual, and ethical development that our complex national and global problems require" (p. 3), the development of these credit-bearing positive youth development courses are timely responses. It is our humble wish that our experience in Hong Kong can serve as a modest example for other colleagues who are passionate about holistic development in university students.
